# The Alteration of Carnitine Metabolism in Second Trimester in GDM and a Nomogram for Predicting Macrosomia

**DOI:** 10.1155/2020/4085757

**Published:** 2020-08-11

**Authors:** Man Sun, Baihui Zhao, Sainan He, Ruopeng Weng, Binqiao Wang, Yunping Ding, Xinwen Huang, Qiong Luo

**Affiliations:** ^1^Department of Obstetrics, Women's Hospital, School of Medicine, Zhejiang University, China; ^2^Department of Genetic and Metabolic Diseases, The Children's Hospital, School of Medicine, Zhejiang University, No.1, Xueshi Road, Shangchen District, Hangzhou, China

## Abstract

**Objective:**

The metabolism of three major nutrients (sugar, lipid, and protein) will change during pregnancy, especially in the second trimester. The present study is aimed at evaluating carnitine alteration in fatty acid metabolism in the second trimester of pregnancy and the correlation between carnitine and GDM.

**Methods:**

450 pregnant women were recruited in the present prospective study. Metabolic profiling of 31 carnitines was detected by LC-MS/MS in these women. Correlation between carnitine metabolism and maternal and neonatal complication with GDM was analyzed.

**Results:**

We found the levels of 7 carnitines increased in age > 35, BMI ≥ 30, weight gain > 20 kg, and ART pregnant groups, but the level of free carnitine (C0) decreased. Nine carnitines were specific metabolites of GDM. Prepregnancy BMI, weight gain, and carnitines (C0, C3, and C16) were independent risk factors associated with GDM and related macrosomia. C0 was negatively correlated with FBG, LDL, TG, and TC. A nomogram was developed for predicting macrosomia in GDM based on carnitine-related metabolic variables.

**Conclusion:**

The carnitine metabolism in the second trimester is abnormal in GDM women. The dysfunction of carnitine metabolism is closely related to the abnormality of blood lipid and glucose in GDM. Carnitine metabolism abnormality could predict macrosomia complicated with GDM.

## 1. Introduction

Pregnancy is a complex process accompanied by substantial changes in sugar, protein, and lipid metabolism [1]. Maternal lipid and protein metabolism of the second trimester is an anabolic state combined with accelerating maternal fat stores and increasing protein synthesis [2]. Dyslipidemia is associated with maternal metabolic disorder (GDM, hyperlipemia, and hypertension) and adverse neonate outcomes (macrosomia, fetal growth restriction) [3]. Fatty acid oxidation decreases in obesity during pregnancy and has linked to GDM development [4].

GDM is a common metabolic disorder during pregnancy and occurs in approximately 10–15% of pregnancies globally [5]. Pregnant women with GDM are more prone to hypertension and metabolic syndrome. The risks to the fetus include macrosomia, respiratory distress syndrome, childhood obesity, and type 2 diabetes mellitus (T2DM) in adults [6]. GDM is associated with profound changes in metabolism. Free carnitine (C0) has a critical role in energy metabolism of transporting long-chain fatty acid from the cytosol into the mitochondria, which results in C0 transforming into acylcarnitine (AC) [7, 8]. Carnitine deficiency is defined as a serum C0 level < 20 *μ*mol/L [9]. C0 deficiency might impair lipid metabolism resulting in GDM [10]. Evaluated circulating AC (such as C3 and C5) is associated with GDM and induces pancreatic *β*-cell dysfunction [11]. A previous study proposed that C0 and AC decreased in pregnancy in the first trimester compared with nonpregnancy [12]. However, studies of metabolic profiling of carnitine in the second trimester among diverse pregnant women are rare. Herein, we provide a study of the metabolism of carnitine to investigate the potential risk factors in GDM.

In the present study, a LC–MS/MS-based metabolomics approach was used to quantify the maternal plasma level of AC in order to identify the correlation between nonglucose metabolism abnormalities and pregnancy metabolic diseases during the second trimester. We analyzed metabolic alteration in the second trimester and detected the independent risk factors of GDM. Moreover, we established a prognostic nomogram of incorporating AC for predicting GDM macrosomia.

## 2. Method

### 2.1. Subject

This study was a prospective study. Between June 2017 and April 2018, 450 pregnant women in the second trimester were recruited in this study at Women's Hospital, School of Medicine, Zhejiang University, in Hangzhou. The study design has been approved by the Ethics Committee of the hospital. All participants were of Chinese Han ethnicity. We obtain maternal characteristic information as follows: gravidity, parity, age, height, prepregnancy BMI, weight gain during pregnancy, use of assisted reproductive technique (ART), and method of pregnancy termination. The following information of the offspring was collected: termination of pregnancy weeks, sex, birth length, actual birth weight, head circumference, abdomen circumference, and abdomen minus head circumference (data from fetal growth measurement ultrasound before labor).

GDM was defined according to the Chinese Current Care Guidelines for GDM as one or more pathological glucose values in a standard oral glucose tolerance test (OGTT) [13]. The diagnostic thresholds were fasting plasma glucose: 5.1 mmol/L, 1 h 10.0 mmol/L, and 2 h 8.5 mmol/L [14]. According to the guideline, 64 GDM cases were diagnosed. The control group was composed of 128 randomly sampled women without GDM from residual pregnant women participating in this study matched by birthday, age, delivery mode, and number of fetuses with 2 : 1 ratio. The 2 : 1 design can eliminate the interference of many factors (such as age, height, and gestational week at delivery) in the study, and a double sample has good statistical performance. The common definition of macrosomia is birth weight over 4000 g [15]. Biochemical status of the GDM and control groups was also collected and analyzed.

### 2.2. Metabolic Profiling Detection by LC-MS/MS

We used the method of LC-MS/MS to investigate the level of 31 plasma carnitines of pregnant women in the second trimester. We also obtain their neonate blood plasma sample. Blood samples were taken via venipuncture, using 4 mL Vacutainer Tubes containing K2-EDTA as anticoagulant centrifuged at 2500 g for 15 min; then, samples of plasma were stored at – 20°C and detected monthly. For plasma analysis, plasma was pipetted into a 2.0 mL 96-deep-well plate. To this, internal standard solution was added, and the plate was vortex mixed for 1 min. Proteins were precipitated by adding 0.3 M zinc sulfate in methanol (1 : 5 *v*/*v*), and the plate was vortexed again for 1 min. Water was added, and the plate was sealed and centrifuged. Following centrifugation, supernatant was injected for analysis [16]. Furthermore, sample preparation used in tandem mass spectrometry (4000 QTrap™; AB Sciex, Darmstadt, Germany) test the concentration. The method used in the present study was essentially a modification of the procedure described elsewhere [17]. AA and AC were quantified using appropriate isotope-labelled standards. LC separation was performed on an Acquity UPLC HSS T3 column (2.1∗100 mm, 100 Å, 1.8 lm particle size; Waters Corporation, MA) using water with 0.1% formic acid, 5 mM ammonium acetate, and 0.015% heptafluorobutyric acid as solvent A and methanol with 0.1% formic acid and 5 mM detected with a Xevo-G2-QTOF MS (Waters Corporation) operating in a positive mode. Raw data was processed using Targetlynx as described previously. Accuracy of quantification was below 6% for all quantified metabolites except glutamic acid (13.9%). Quantitative data was obtained using MetIDQTM Software [18].

### 2.3. Statistical Analysis

Data distributed normally were expressed as means ± standard deviation. The statistical method used for testing difference between two groups was Student's*t*-test. One-way ANOVA was used in more than two groups. For correlation analysis, the Spearman correlation coefficient was calculated in the level of AA and AC between the mothers in the second trimester and their neonates. In PLS-DA, metabolomics data were log-transformed to ensure a normally distributed data set; *R*^2^ (goodness of fitness) and *Q*^2^ (goodness of prediction) were assessed in the PLS-DA models. Multivariate logistic regression analysis was used to determine the association of GDM and macrosomia with prepregnancy BMI, weight gain, and ACs. Odds ratios (ORs) and 95% confidence intervals (CIs) were reported per standard deviation. All statistical analysis was performed using IBM SPSS 23.0 edition, SIMCA 14.0, and R vision 3.6.0. A significance level of 0.05 was used for all statistical tests.

## 3. Results

**Figure 1:**
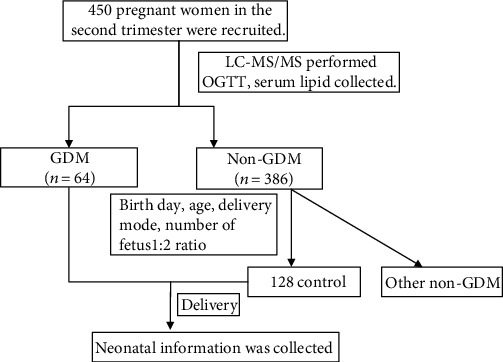
Study workflow of the present prospective study.

The study setup is illustrated in Figure 1. We identified the metabolic alternation in 450 pregnant women of the second trimester. We also investigated the metabolic risk factors in GDM and established a prognostic nomogram based on AC risk factors for predicting macrosomia.

### 3.1. Maternal Plasma AC Levels of the Second Trimester in 450 Pregnant Women

**Figure 2:**
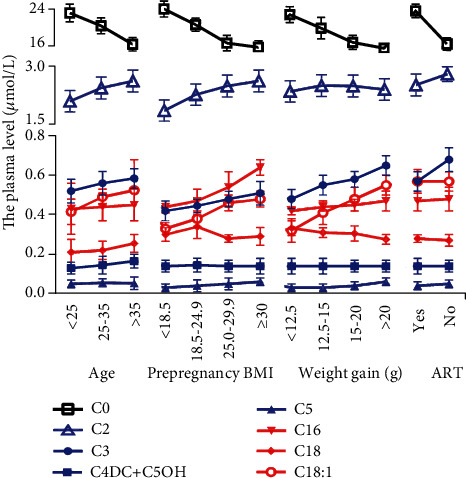
The maternal plasma levels of acylcarnitine (AC) at the second trimester in 450 pregnant women according to clinical characteristic. The AC level was higher in the age > 35 group, in prepregnancy BMI ≥ 25.0 and 30, in the weight gain group, and in the ART group, while C0 was decreased. Values are presented as the mean ± SEM.

The baseline characteristics of 450 study participants divided into different categories according to natural conditions of pregnant women such as gravidity, parity, age, height, prepregnancy BMI, weight gain, ART, and method of pregnancy termination. The statistically significant results are shown in Table 1 and Figure 2. In the age subgroup, there was a trend that the level of AC (C2, C3, C4DC+C5OH, C16, C18, and C18:1) was higher in the age > 35 group, whereas the level of C0 in the age > 35 group was lower. In prepregnancy BMI ≥ 25.0 and 30 subgroups, several ACs (C2, C3, C5, C16, and C18:1) were higher, whereas the level of C0 was lower. In the weight gain group, C3, C5, C16, and C18:1 were higher in the weight gain ≥ 20 kg group than the other groups, while C0 was lower. In the ART group, C2, C3, and C5 were higher while C0 was lower. There was no statistical difference in gravity, parity, height, and method of pregnancy termination subgroups (data not shown).

**Figure 3:**
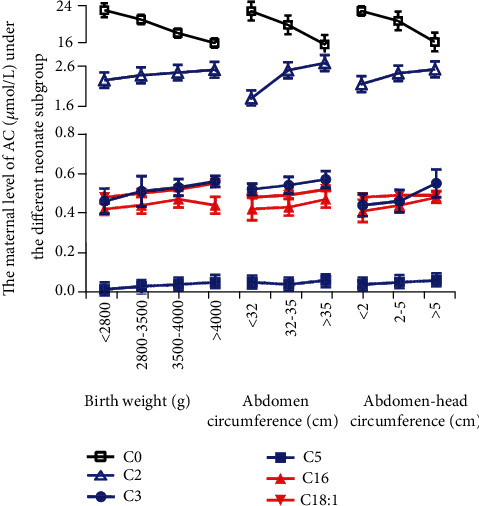
The maternal plasma levels of acylcarnitine (AC) at the second trimester in 450 pregnant women according to different neonate subgroups. The AC level was higher in the birth weight > 4000 g group, in the abdomen circumference > 35 cm group, and in the abdomen minus circumference > 5 subgroup. Values are presented as the mean ± SEM.

Additionally, we investigated the maternal level AC in second trimester pregnancy under the different neonate subgroups (termination of pregnancy weeks, sex, birth length, birth weight, head circumference, abdomen circumference, and abdomen minus head circumference). The results are shown in Table 2 and Figure 3. In the birth weight > 4000 g group, we found that ACs (C2, C3, C5, C16, and C18:1) were higher; these metabolites increased with birth weight, while C0 was lower. In the abdomen circumference > 35 cm group, several ACs (C2, C3, C5, and C16) were higher; C0 was lower. In the abdomen minus circumference subgroup, the metabolite characteristics have a similar trend with the abdomen circumference > 35 cm subgroup.

### 3.2. Specific AC Distribution in GDM

**Figure 4:**
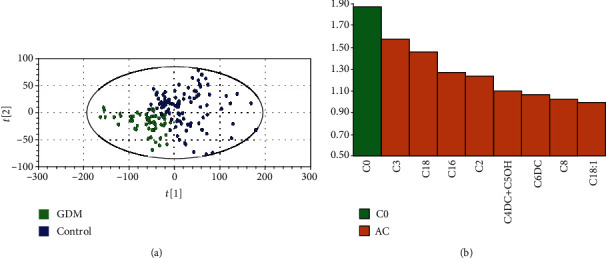
Specific acylcarnitine (AC) distribution in GDM. (a) Partial least squares discrimination analysis (PLS-DA). Class separation is shown by loading plot. (b) Variable importance in prediction (VIP).

We examined the serum metabolite in 64 GDM patients and 128 matched patients without GDM. Our study used the PLS-DA model (*R*^2^ = 0.527, *Q*^2^ = 0.464) to analyze differences between two groups. The PLS-DA scatterplot showed a clear class separation with GDM at the left and the control group at the right (Figure 4(a)). Furthermore, we used variable importance in projection (VIP) to estimate the contribution of every AC to class separation (GDM vs. control). A VIP value > 1.0 was considered with a high contribution to class separation. The VIP analysis (Figure 4(b)) showed that C0 (VIP = 1.87) plays the main role in class separation.

### 3.3. Clinical Characteristics and AC Plasma Level of GDM

Table 3 shows the characteristics of GDM patients and control. The prepregnancy BMI, weight gain, ART, and the serum levels of C2, C3, C4DC+C5OH, C6DC, C8, C16, C18, and C18:1 were higher, while those of C0 was lower in the GDM group.

### 3.4. Multiple Logistic Regression Analysis of the Association between GDM and Other Factors

We selected significant factors from univariate analysis to enter multiple logistic regression analysis to examine whether acting independently. Prepregnancy BMI (OR = 1.15, 95%CI = 1.06‐1.78), weight gain (OR = 1.18, 95%CI = 1.03‐1.64), C0 (OR = 0.70, 95%CI = 0.60‐0.83), C3 (OR = 1.03, 95%CI = 1.02‐2.08), C16 (OR = 1.30, 95%CI = 1.12‐3.28), and C18 (OR = 1.27, 95%CI = 1.00‐3.01) were statistically associated with GDM. These factors can work as independent risk factors involve in the process of GDM (Table 4).

### 3.5. Association of C0 Deficiency and Blood Glucose and Lipid in GDM and Control

**Figure 5:**
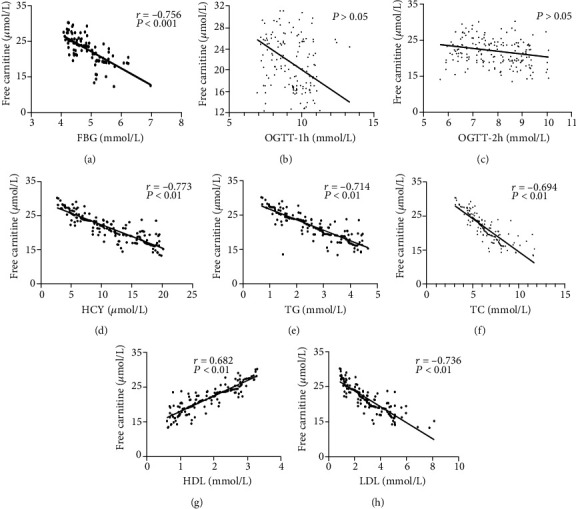
The relationship between C0 and blood glucose and lipid in GDM (*n* = 64) and non-GDM (*n* = 128) groups. The correlation between C0 and FBG (a), OGTT-1 h (b), OGTT-2 h (c), HCY (d), TG (e), TC (f), HDL (g), and LDL (h). *r* = Spearman's correlation coefficient.

When divided into two groups according to the C0 level (<20 *μ*mol/L or ≥20 *μ*mol/L), we found that 81.25% of GDM women of the second trimester pregnancy are in C0 deficiency status. In GDM women, FBG, TG, TC, LDL, and homocysteine (HCY) were significantly higher in the C0 < 20 *μ*mol/L group. Similar results were also seen in the control group of C0 < 20 *μ*mol/L (Table 5). In GDM and control groups, C0 was negatively correlated with FBG, LDL, TG, TC, and HCY and positively correlated with HDL. There were no significant correlations between C0 and OGTT-1 h and OGTT-2 h (Figure 5).

### 3.6. Cluster Correlation Heat Map

**Figure 6:**
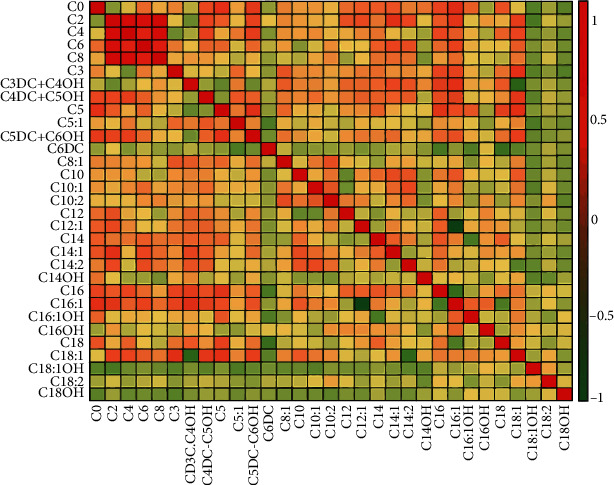
Correlation heat map of all targeted metabolites. In red is the cluster of strongly intercorrelated metabolites.

The heat map depicting the internal correlations between metabolites revealed the predominant clusters of intercorrelated metabolites: C2, C4, C6, and C8 (Figure 6). The four metabolites included in the final model showed strongly internal correlations. There were no strong intercorrelations in others.

### 3.7. Clinical Characteristics and AC Plasma Level and a Nomogram of Prediction of Macrosomia

**Figure 7:**
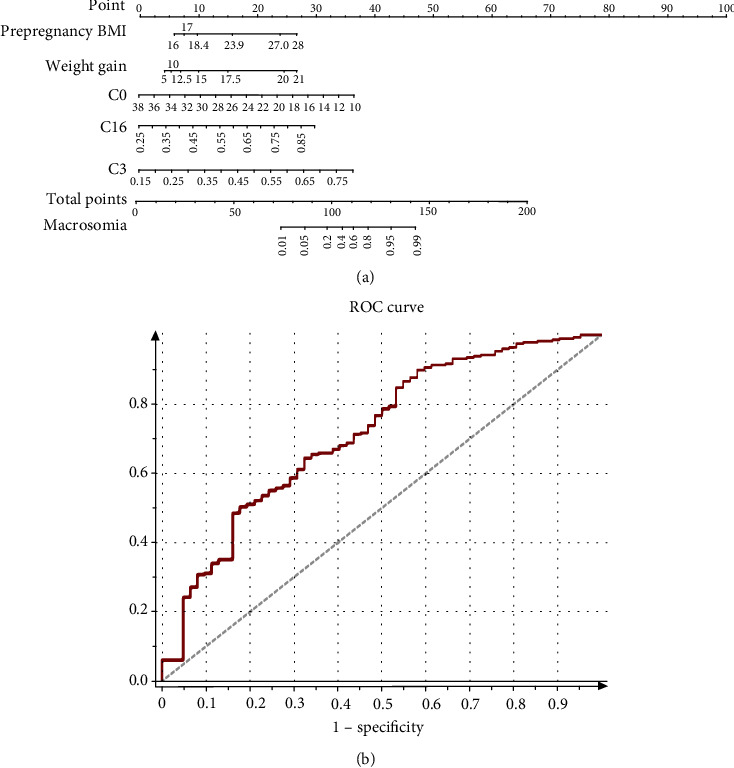
Predictive graphic nomogram for probability of GDM macrosomia and ROC curve. (a) Prepregnancy BMI, weight gain, C0, C3, and C16-based nomogram for predicting macrosomia, predictor points (points^ scale; top) correspond to each variable. Points for all these variables are added and translated into the probability of macrosomia in GDM. (b) Receiver operating characteristic (ROC) curves for validated models. AUC = 0.88.

Here, we investigated the clinical characteristics and AC metabolite between GDM with macrosomia and GDM without macrosomia (Table 6). We found that prepregnancy weight, BMI, weight gain, C0, C2, C3, C16, and C18 were higher in GDM with macrosomia (*P* < 0.05). In the multiple logistic regression analysis, we found that prepregnancy BMI, weight gain, C0, C3, and C16 were also evaluated (Table 7, *P* < 0.05). These factors are independent risk factors involved in the process of GDM-induced macrosomia. The nomogram of predicting GDM-induced macrosomia had incorporated these significant variables. Among these metabolites, C0 deficiency showed the highest OR (OR = 0.75, 95%CI = 0.50‐0.87). Vertical lines should be drawn from the correct location from each independent risk factor. “Total points” which could be obtained by adding all points of the axis to the bottom axes made the conversion into a macrosomia probability. The accuracy of the model was well assessed by the AUC equal to 0.78 (Figure 7).

## 4. Discussion

In the present study, we have identified metabolic alteration in the second trimester of pregnancy by LC-MS/MS. We found that 9 carnitines including free carnitine (C0) were significantly related to GDM. Combined with clinical information, multivariable logistic regression had demonstrated that prepregnancy BMI, weight gain, C0, C3, C16, and C18 were independent metabolic risk factors associated with GDM. C0 played a vital role in GDM and GDM-complicated macrosomia. C0 deficiency was significantly related to GDM, and abnormal metabolism of blood glucose and lipid was accompanied by GDM. We also had developed a nomogram to predict probability of macrosomia with GDM based on C0-related metabolites. To our knowledge, this is the first study reporting carnitine alteration during the second trimester of pregnancy and abnormal metabolism of carnitine in GDM and macrosomia with GDM.

Carnitine metabolism is an important part of fatty acid metabolism. Several experimental works have indicated characteristics of carnitine-related metabolites during pregnancy [12, 19, 20]. Total carnitine consisted of C0 and AC. C0 is required for fatty acid transfer into the mitochondrial membrane; in this process, the ester carnitines are formed. Ester carnitine presented as AC releasing into plasma [21].

An interesting finding appears that there is a pronounced fall of the plasma content of C0, AC, and total carnitine during pregnancy [22]. In our study, we found that some carnitines (C0, C2, C3, C4DC+C5OH, C5, C16, C18, and C18:1) increased in the age > 35, BMI ≥ 30, and weight gain > 20 kg groups. However, C0 was decreased in these groups. These results were similar with the findings of a study by Fujiwara et al. which demonstrated that AC was accumulated in obesity of hepatocellular carcinoma [23]. The plasma AC accumulation suggested an incomplete long-chain fatty acid oxidation and altered tricarboxylic acid activity. Oxidative stress associated with AC accumulation is likely responsible for insulin resistance and diabetes. The study demonstrated that long-term AC accumulation was a feature of T2DM [24]. C0 may increase fatty acid *β*-oxidation and basal metabolic rates. Other studies had proved that the oxidation rate of fatty acids gradually decreased with age [25].

GDM is defined as glucose intolerance dysfunction during pregnancy. The prevalence of GDM increased from 5% to 14% in USA [26]. The plasma carnitine concentrations show a strong correlation with the development of diabetes [27]. In our present study, C0 could be confirmed as the potential risk factor in GDM. A study by Batchuluun et al. stated that evaluated AC was associated with GDM through impairing insulin synthesis [28]. Our study also found that serum concentration of some AC significantly increased in the GDM group. A research by Hansen et al. reported that excess of AC in mitochondria might be harmful for metabolism dynamic balance due to aerobic glycolysis disturbance [29]. Previous studies suggested that accumulation of AC affected glucose and lipid metabolism in GDM [10, 28]. An interesting finding by Yau et al. reported that AC might impair mammalian insulin signal transduction through acting at mTOR phosphorylation [30].

Lack of C0 may elevate the lipid level and further results in GDM [31]. A meta-analysis by Asadi et al. showed a significant relationship between C0 and TG, TC, LDL, and HDL in adults with cardiovascular risk factors. The study indicated a significant effect of C0 supplement (1500 mg/d) on lowering serum levels of TG, TC, and LDL in atherosclerosis patients [32]. Our study reported that serum C0 concentration was negatively correlated with FBG, LDL, TG, TC, and HCY and positively correlated with HDL especially in the GDM group, which is consistent with previous researches [33]. In Nowak et al.'s study, C0 showed no association with insulin resistance during OGTT, and C10 and C12 decreased during OGTT with worse insulin resistance as well [34]. Our present study confirmed that C0 was significantly related to FBG but not 1 h and 2 h during OGTT. It is widely accepted that FBG represented the severity of GDM. Our results confirmed that C0 deficiency was significantly related to the abnormal metabolism of glucose and lipid in the second trimester of pregnancy with GDM.

Macrosomia is associated with an increased risk of neonatal morbidity and obesity in adult [35]. GDM is associated with an increasing risk of macrosomia [36]. Many studies have proved that carnitine deficiency is associated with lipid metabolism in GDM [37, 38]. So, we speculated that carnitine is involved in macrosomia with GDM. C0 supplement is effective in normalizing insulin sensitivity of GDM and controlling the synthesis of key glycolytic and gluconeogenic enzymes. Macrosomia is regarded as a disorder of energy balance, which perturbs body weight homeostasis [39]. Antimacrosomia effect of C0 supplement might be mediated by the induction of lipolysis and fatty acid oxidation. Our study confirmed that C0 is a protective factor for macrosomia. However, C3 and C16 were risk factors for macrosomia with GDM. Therefore, the characteristics of carnitine metabolism had enabled us to discover reliable biomarkers and set up a nomogram model to predicting GDM-related macrosomia.

Our study has several limitations. We should expand the sample size and detect more metabolites to clarify the significance of nonglycometabolism during pregnancy. Also, the metabolites in the other trimesters such as the first trimester should be detected for predicting the occurrence of GDM by carnitine or other metabolites.

## 5. Conclusions

In conclusion, by analyzing the metabolic alteration in the second trimester, we found abnormal metabolism of carnitine including C0, C3, C16, and C18 which are independent risk factors of GDM. C0 deficiency during pregnancy is significantly obvious in GDM and closely related to the abnormality of blood lipid and glucose of GDM. Carnitine metabolism abnormality could predict macrosomia complicated with GDM. In all, the abnormality of fatty acid metabolism is of great significance in the pathogenesis and the maternal and neonatal complications of GDM.

## Figures and Tables

**Table 1:** The maternal plasma levels of acylcarnitine (AC) at the second trimester in 450 pregnant women according to clinical characteristic. Values are presented as the mean ± SD; ^∗^*P* < 0.05 is considered statistically significant.

	Age			*P* value	Prepregnancy BMI				*P* value	Weight gain (kg)				*P* value	ART		*P* value
	<25	25-35	>35		<18.5	18.5-24.9	25.0-29.9	≥30		<12.5	12.5-15	15-20	>20		No	Yes	
*n*	12	247	191		9	253	153	35		97	147	150	56		376	74	
C0	23.26 ± 3.86	20.34 ± 3.81	16.18 ± 7.55	0.035^∗^	24.15 ± 3.8	20.63 ± 5.49	16.43 ± 4.76	15.52 ± 3.13	0.038^∗^	22.85 ± 3.8	19.83 ± 5.45	16.63 ± 4.62	15.28 ± 3.14	0.016^∗^	23.71 ± 5.54	16.25 ± 7.43	0.002^∗^
C2	2.08 ± 0.54	2.43 ± 0.76	2.61 ± 0.92	0.043^∗^	1.84 ± 0.49	2.25 ± 0.82	2.48 ± 0.78	2.61 ± 0.67	0.017^∗^	2.34 ± 0.42	2.49 ± 0.56	2.48 ± 0.28	2.39 ± 0.67	0.640	2.51 ± 0.90	2.79 ± 0.82	0.016^∗^
C3	0.52 ± 0.14	0.56 ± 0.17	0.58 ± 0.20	0.042^∗^	0.42 ± 0.14	0.45 ± 0.19	0.48 ± 0.18	0.55 ± 0.17	0.015^∗^	0.48 ± 0.14	0.55 ± 0.19	0.58 ± 0.19	0.65 ± 0.17	0.035^∗^	0.57 ± 0.23	0.68 ± 0.15	0.042^∗^
C4DC+C5OH	0.13 ± 0.03	0.14 ± 0.05	0.16 ± 0.02	0.014^∗^	0.14 ± 0.03	0.14 ± 0.06	0.14 ± 0.04	0.14 ± 0.04	0.290	0.14 ± 0.03	0.14 ± 0.06	0.14 ± 0.02	0.14 ± 0.04	0.490	0.14 ± 0.06	0.14 ± 0.06	0.185
C5	0.05 ± 0.02	0.04 ± 0.01	0.04 ± 0.01	0.524	0.03 ± 0.01	0.04 ± 0.02	0.05 ± 0.01	0.06 ± 0.01	0.027^∗^	0.02 ± 0.01	0.03 ± 0.02	0.04 ± 0.01	0.06 ± 0.01	0.038^∗^	0.03 ± 0.01	0.05 ± 0.01	0.035^∗^
C16	0.43 ± 0.13	0.44 ± 0.17	0.45 ± 0.18	0.019^∗^	0.44 ± 0.13	0.47 ± 0.19	0.54 ± 0.18	0.68 ± 0.28	0.026^∗^	0.42 ± 0.15	0.44 ± 0.19	0.45 ± 0.18	0.47 ± 0.25	0.029^∗^	0.47 ± 0.28	0.48 ± 0.17	0.338
C18	0.26 ± 0.08	0.27 ± 0.11	0.30 ± 0.10	0.032^∗^	0.30 ± 0.09	0.28 ± 0.13	0.28 ± 0.10	0.29 ± 0.17	0.510	0.31 ± 0.09	0.31 ± 0.13	0.29 ± 0.11	0.30 ± 0.17	0.560	0.28 ± 0.13	0.27 ± 0.11	0.483
C18:1	0.48 ± 0.13	0.49 ± 0.16	0.54 ± 0.24	0.017^∗^	0.32 ± 0.12	0.38 ± 0.23	0.46 ± 0.19	0.52 ± 0.17	0.019^∗^	0.32 ± 0.18	0.41 ± 0.26	0.48 ± 0.16	0.55 ± 0.16	0.045^∗^	0.57 ± 0.21	0.57 ± 0.27	0.467

**Table 2:** The maternal plasma levels of acylcarnitine (AC) at the second trimester in 450 pregnant women according to different neonate subgroups. Values are presented as the mean ± SD; ^∗^*P* < 0.05 is considered statistically significant.

	Birth weight (g)				*P* value	Abdomen circumference (cm)			*P* value	Abdomen-head circumference (cm)			*P* value
	<2800	2800-3500	3500-4000 g	>4000 g		<32	32-35	>35		<2	2-5	5	
C0	23.38 ± 5.23	21.37 ± 4.77	18.52 ± 3.13	16.40 ± 4.74	0.026^∗^	23.15 ± 3.80	20.22 ± 5.18	16.11 ± 4.04	0.018^∗^	23.19 ± 5.16	21.08 ± 4.06	16.61 ± 3.14	0.014^∗^
C2	2.30 ± 0.82	2.42 ± 0.78	2.49 ± 0.67	2.56 ± 0.78	0.023^∗^	1.84 ± 0.49	2.55 ± 0.82	2.73 ± 0.76	0.023^∗^	2.20 ± 0.82	2.47 ± 0.76	2.57 ± 0.66	0.033^∗^
C3	0.46 ± 0.18	0.51 ± 0.16	0.53 ± 0.17	0.56 ± 0.18	0.014^∗^	0.52 ± 0.14	0.54 ± 0.16	0.57 ± 0.18	0.024^∗^	0.44 ± 0.164	0.51 ± 0.18	0.55 ± 0.17	0.013^∗^
C5	0.02 ± 0.01	0.03 ± 0.02	0.04 ± 0.01	0.05 ± 0.01	0.037^∗^	0.05 ± 0.02	0.04 ± 0.01	0.06 ± 0.01	0.025^∗^	0.04 ± 0.02	0.05 ± 0.01	0.06 ± 0.01	0.026^∗^
C16	0.42 ± 0.12	0.44 ± 0.18	0.47 ± 0.20	0.44 ± 0.16	0.027^∗^	0.42 ± 0.10	0.43 ± 0.12	0.47 ± 0.17	0.026^∗^	0.41 ± 0.12	0.44 ± 0.18	0.48 ± 0.20	0.015^∗^
C18:1	0.48 ± 0.15	0.50 ± 0.17	0.52 ± 0.17	0.55 ± 0.14	0.017^∗^	0.48 ± 0.12	0.49 ± 0.15	0.52 ± 0.16	0.207	0.48 ± 0.15	0.49 ± 0.16	0.49 ± 0.18	0.123

**Table 3:** Clinical characteristics and ACs of women with GDM and matched control women. Values are presented as the mean ± SD for continuous variables and percentage for dichotomous variables. Cases and controls were matched for birth day, age, delivery mode, and number of fetuses with a 2 : 1 ratio. ^∗^*P* value for Student's *t*-test (continuous variables) or chi-squared test (dichotomous variables).

	GDM	Control	*P* value
Total	64	128	
Maternal age (years)	34.32 ± 3.42	32.41 ± 3.65	0.28
Height (cm)	160.34 ± 5.45	161.23 ± 4.45	0.35
Prepregnancy weight (kg)	60.52 ± 4.53	64.45 ± 3.69	0.21
Prepregnancy BMI (kg/m^2^)	23.82 ± 3.42	21.25 ± 2.32	0.03^∗^
Weight gain (kg)	22.53 ± 4.37	17.23 ± 2.89	0.02^∗^
Gestational week at delivery (weeks)	38.32 ± 0.67	39.45 ± 0.72	0.54
Nulliparous	76.40	80.20	0.64
ART	19.6	14.2	0.04
Smoker	7.1	8.0	0.35
Birth weight (g)	3847.23 ± 100.78	3304.56 ± 92.65	0.03^∗^
C0 (*μ*mol/L)	16.66 ± 3.37	24.12 ± 8.34	0.03^∗^
C2	3.60 ± 0.77	3.06 ± 1.08	0.03^∗^
C3	0.54 ± 0.16	0.63 ± 0.29	0.02^∗^
C4DC+C5OH	0.13 ± 0.04	0.16 ± 0.07	0.01^∗^
C6DC	0.06 ± 0.01	0.04 ± 0.01	0.03^∗^
C8	0.07 ± 0.04	0.04 ± 0.02	0.04^∗^
C16	0.84 ± 0.14	0.46 ± 0.20	0.04^∗^
C18	0.47 ± 0.09	0.28 ± 0.14	0.02^∗^
C18:1	0.78 ± 0.16	0.50 ± 0.24	0.04^∗^

**Table 4:** Multiple logistic regression analysis of the association between GDM and other factors. OR: odds ratio; 95% CI: 95% confidence intervals.

	OR	95% CI	*P* value
Prepregnancy BMI (kg/m^2^)	1.15	1.06-1.78	0.03^∗^
Weight gain (kg)	1.18	1.03-1.64	0.03^∗^
ART	1.24	1.00-1.39	0.73
C0 (*μ*mol/L)	0.70	0.60-0.83	0.02^∗^
C2	1.32	0.65-4.37	0.32
C3	1.03	1.02-2.08	0.01^∗^
C4DC+C5OH	1.01	1.01-2.48	0.81
C6DC	1.93	1.20-3.72	0.93
C8	1.26	1.10-4.35	0.56
C16	1.30	1.12-3.28	0.03^∗^
C18	1.27	1.00-3.01	0.04^∗^
C18:1	1.17	1.13-2.76	0.17

**Table 5:** The association of C0 deficiencies and maternal lipid and glucose in GDM and non-GDM groups. Values are presented as the mean ± SD for continuous variables and percentage for dichotomous variables. ^∗^*P* value for Student's *t*-test.

	GDM		*P* value	Control		*P* value
	C0 < 20	C0 ≥ 20		C0 < 20	C0 ≥ 20	
*n*	52 (81.25%)	12 (128.75)		96 (75%)	32 (25%)	
FBG (mmol/L)	5.62 ± 0.82	5.19 ± 0.63	0.02^∗^	5.03 ± 0.65	4.65 ± 0.32	0.03^∗^
OGTT-1 h (mmol/L)	10.12 ± 0.29	10.14 ± 0.26	0.43	9.84 ± 0.44	9.43 ± 0.51	0.36
OGTT-2 h (mmol/L)	8.67 ± 0.45	8.59 ± 0.41	0.39	8.35 ± 0.26	7.92 ± 0.33	0.28
TG (mmol/L)	3.12 ± 0.32	1.64 ± 0.25	0.02^∗^	3.02 ± 0.27	1.94 ± 0.21	0.02^∗^
TC (mmol/L)	6.32 ± 0.45	4.43 ± 0.62	0.03^∗^	5.18 ± 0.62	4.32 ± 0.32	0.03^∗^
HDL (mmol/L)	1.78 ± 0.26	1.83 ± 0.31	0.39	1.71 ± 0.13	1.92 ± 0.28	0.04^∗^
LDL (mmol/L)	4.89 ± 0.82	3.16 ± 0.76	0.02^∗^	4.63 ± 0.21	3.04 ± 0.65	0.02^∗^
HCY (*μ*mol/L)	17.67 ± 1.02	12.31 ± 1.11	0.03^∗^	15.32 ± 0.64	11.01 ± 0.82	0.03^∗^

**Table 6:** Clinical characteristics and ACs of GDM with macrosomia and nonmacrosomia. Values are presented as the mean ± SD for continuous variables and percentage for dichotomous variables. ^∗^*P* value for Student's *t*-test (continuous variables) or chi-squared test (dichotomous variables).

	Macrosomia	Nonmacrosomia	*P* value
*n*	12	52	
Maternal age (years)	34.98 ± 4.68	32.23 ± 4.43	0.79
Height (cm)	159.46 ± 5.94	161.46 ± 5.95	0.33
Prepregnancy weight (kg)	56.25 ± 9.98	52.42 ± 7.84	0.04^∗^
Prepregnancy BMI (kg/m^2^)	24.55 ± 4.15	22.21 ± 3.02	0.03^∗^
Weight gain (kg)	24.07 ± 4.11	18.42 ± 4.10	0.02^∗^
Gestational week at delivery (weeks)	38.4 ± 0.78	39.6 ± 0.97	0.83
Nulliparous (%)	74.2	62.5	0.82
ART	19.6	14.2	0.55
Smoker	7.4	8.2	0.96
Birth weight (g)	4247.52 ± 100.78	3597.34 ± 98.65	0.02^∗^
C0 (*μ*mol/L)	10.68 ± 3.28	17.12 ± 5.66	0.03^∗^
C2	3.64 ± 0.76	3.58 ± 1.25	0.03^∗^
C3	0.43 ± 0.17	0.64 ± 0.26	0.02^∗^
C4DC+C5OH	0.12 ± 0.04	0.14 ± 0.05	0.05
C6DC	0.06 ± 0.01	0.06 ± 0.01	0.77
C8	0.08 ± 0.04	0.07 ± 0.01	0.54
C16	0.86 ± 0.12	0.84 ± 0.20	0.03^∗^
C18	0.50 ± 0.09	0.47 ± 0.13	0.04^∗^
C18:1	0.52 ± 0.16	0.48 ± 0.24	0.55

**Table 7:** Multiple logistic regression analysis of factors associated with GDM macrosomia. OR: odds ratio; 95% CI: 95% confidence intervals.

	OR	95% CI	*P* value
Prepregnancy weight (kg)	1.28	0.76-1.98	0.64
Prepregnancy BMI (kg/m^2^)	1.27	1.02-2.37	0.03^∗^
Weight gain (kg)	1.24	1.46-3.45	0.04^∗^
C0	0.75	0.50-0.87	0.02^∗^
C2	1.15	1.02-3.37	0.68
C3	1.16	1.12-1.98	0.03^∗^
C16	1.15	1.03-1.22	0.04^∗^
C18	1.17	1.24-3.23	0.97

## Data Availability

The datasets used and analyzed during the current study are available from the corresponding author on reasonable request.
